# Supplementary material for: Versatile live-cell activity analysis platform for characterization of neuronal dynamics at single-cell and network level
Source: Nat Commun. 2020 Sep 25;11:4854. doi: 10.1038/s41467-020-18620-4 (PMC7519655; doi:10.1038/s41467-020-18620-4)
Supplement: Supplementary file 1 — Supplementary Information [file 41467_2020_18620_MOESM1_ESM.pdf]

Supplementary Information

Versatile live-cell activity analysis platform for characterization of neuronal dynamics at single-cell and network level

Yuan et al.

Supplementary Figure 1

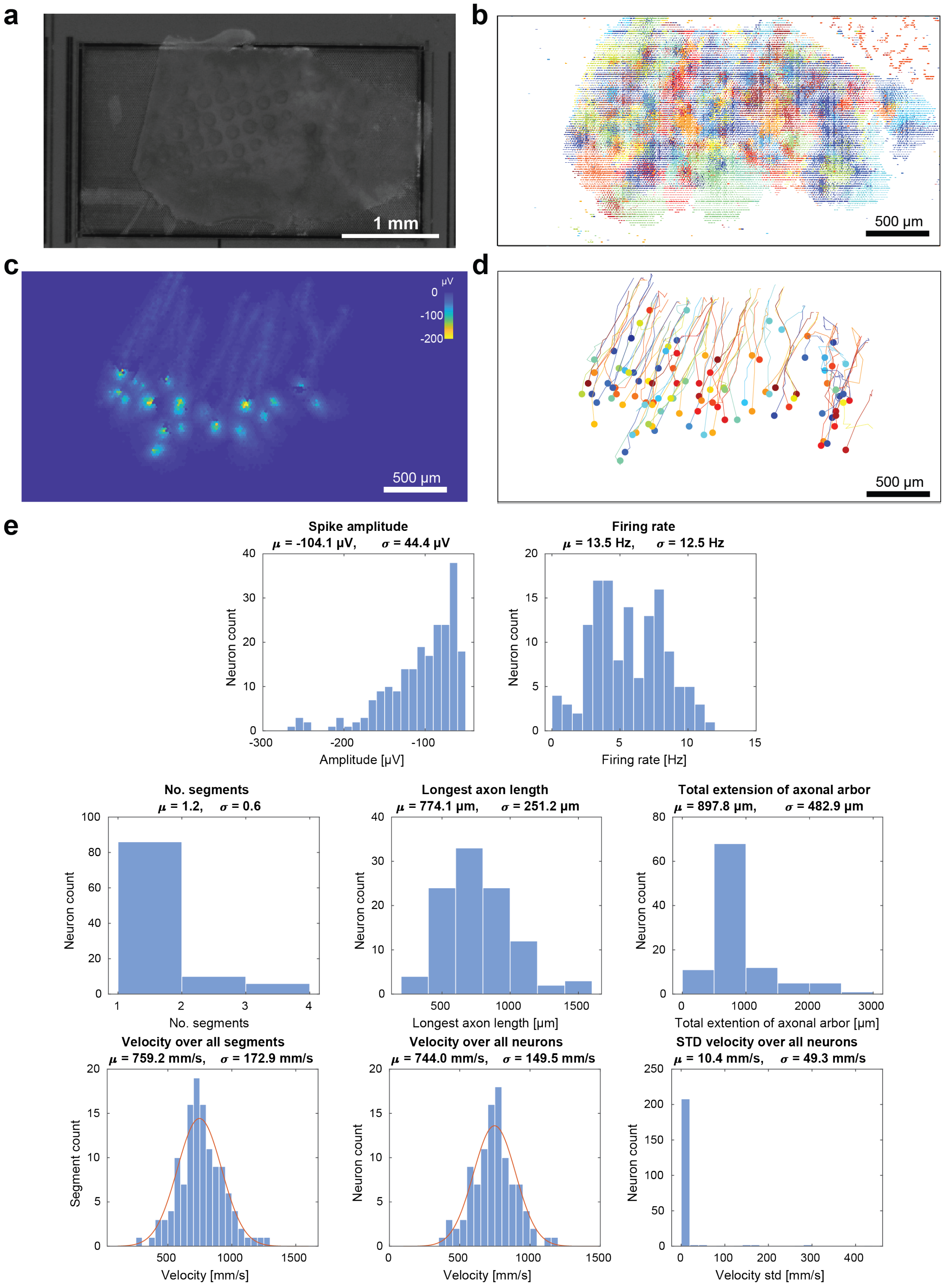

Supplementary Figure 1. Extraction of axonal signal propagation characteristics from the retina recordings. (a) Microscopy image of a retina placed on the DM-MEA. (b) Extracted electrical footprints for n=202 spike-sorted neurons, showing a complete coverage of the retina. (c) Example footprints of 20 neurons. (d) Extracted axonal segments for all detected neurons, showing one axon originating from each neuron. All axons are oriented in the same direction. (e) Statistics extracted for the retina (n = 202 neurons) with the same methods that have been used to obtain the panels in Fig 3, including spike amplitude and firing rate for each neuron, axon lengths and propagation velocities. Mean values (μ) are given as well as standard deviations (σ).

Supplementary Figure 2

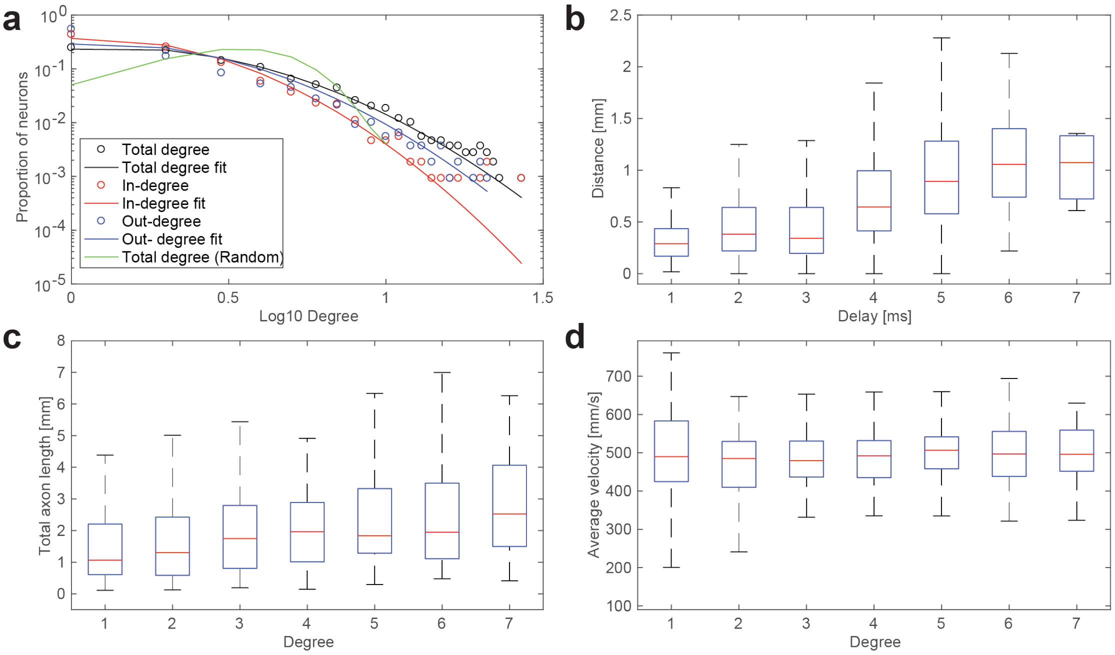

Supplementary Figure 2. Analysis of network connectivity, and correlation between single-cell properties and network connectivity. (a) Degree distribution (in-degree, out-degree and total degree) with log-normal fitting and comparison to random directional network. (b) Correlation between connection length and time delay. (c) Correlation between total degree and total extension of axonal arbor. (d) Correlation between total degree and average velocity. The box plots display the 25th percentile, median, and 75th percentile, and the whiskers represent ± 1.5 × interquartile range.

Supplementary Figure 3

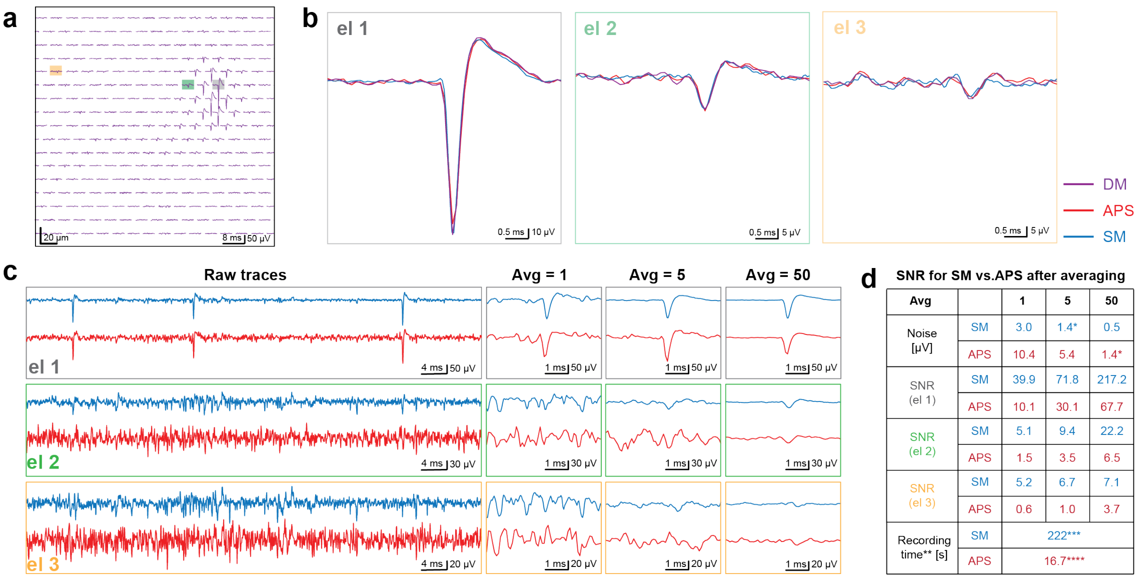

SNR for SM vs.APS after averaging

Avg		1	5	50
Noise [µV]	SM	3.0	1.4*	0.5
	APS	10.4	5.4	1.4*
SNR (el 1)	SM	39.9	71.8	217.2
	APS	10.1	30.1	67.7
SNR (el 2)	SM	5.1	9.4	22.2
	APS	1.5	3.5	6.5
SNR (el 3)	SM	5.2	6.7	7.1
	APS	0.6	1.0	3.7
Recording time** [s]	SM	222***		
	APS	16.7****		

* The noise level for SM mode with averaging 5 APs is 1.4 µV, which is identical to that of the APS mode averaging 50 APs. These numbers and noise values were used for the calculation of recording time at the bottom of the table.
** Recording time required to collect footprints from 100 neurons with an assumed minimum firing rate of 3 Hz.
*** For the SM mode, we assumed that 100 channels were used to record from 100 defined neurons, while the other channels were used to scan through the array applying 134 configurations of 1.67 s duration per configuration to record 5 spikes for spike-triggered averaging to achieve a noise level of 1.4 µV.
**** For the APS mode, one full-frame recording of 16.7 s duration was necessary to record 50 spikes for spike-triggered averaging to achieve the same noise level of 1.4 µV.

Supplementary Figure 3. Comparison of signal averaging results using the different modes (DM, APS and SM). (a) Electrical footprints of one neuron acquired using the DM mode. (b) Averaged waveforms of three selected electrodes (el 1-3, colored background in panel a) acquired using the three different modes (DM, APS and SM). DM: averaging of APS data with triggers from SM data (67 spikes); APS: averaging of APS data with triggers from APS data (66 spikes); SM: averaging of SM data with triggers from SM data (67 spikes). The waveforms are almost identical, while there are differences in noise levels and signal-to-noise (SNR) characteristics (1.34 µV for DM with 67 averaged APs, 1.36 µV for APS with 66 averaged APs, and 0.44 µV for SM with 67 averaged APs). (c) Effect of averaging for the three selected electrodes, showing both, raw data and averaged waveforms with different numbers of averaged APs (1, 5, 50). (d) Summary of noise levels and SNR for SM and APS mode with different numbers of averaged APs. The noise levels were measured and averaged over multiple active electrodes (100 electrodes for APS, 50 electrodes for SM). Since DM and APS both use APS data for averaging, the SNR values with different number of averaged APs for the DM mode are identical to those of the APS mode. In addition, we also estimated the recording time needed to collect footprints from 100 neurons using APS and SM modes.
