# Supplementary material for: Versatile live-cell activity analysis platform for characterization of neuronal dynamics at single-cell and network level
Source: Nat Commun. 2020 Sep 25;11:4854. doi: 10.1038/s41467-020-18620-4 (PMC7519655; doi:10.1038/s41467-020-18620-4)
Supplement: Supplementary file 3 — Description of Additional Supplementary Files [file 41467_2020_18620_MOESM3_ESM.pdf]

Title: Supplementary movie 1.

Description: The video displays raw data (non-averaged, band-pass filtered signals: 0.3-3 kHz), obtained by full-frame recording from a primary rat neuron culture (DIV 20), showing spontaneous electrical activity of developing neurons across the entire array.

Title: Supplementary movie 2.

Description: The video displays raw data (non-averaged, band-pass filtered signals: 1-300 Hz), obtained by full-frame recording from an acute hippocampal slice of a 3-week old mouse, showing signal propagation from the CA3 to CA1 region.

Title: Supplementary movie 3.

Description: The video displays the raw data (non-averaged, band-pass filtered signals: 0.3-3 kHz) obtained by full-frame recording from an excised piece of a retina of an adult Wistar rat.

Title: Supplementary movie 4.

Description: The video displays the raw data (non-averaged, band-pass filtered signals: 0.3-3 kHz) obtained by full-frame recording from iPSC-derived human dopaminergic neurons, cultured on an SCAD device, showing AP propagation along the membrane structure.

Title: Supplementary movie 5.

Description: The video shows the propagation of an AP through the axonal arbor of a single neuron (DIV 20, n = 3390 spike-triggered averages), shown in Figure 5.
